# Dynamic structural evolution of supported palladium–ceria core–shell catalysts revealed by *in situ* electron microscopy

**DOI:** 10.1038/ncomms8778

**Published:** 2015-07-10

**Authors:** Shuyi Zhang, Chen Chen, Matteo Cargnello, Paolo Fornasiero, Raymond J. Gorte, George W. Graham, Xiaoqing Pan

**Affiliations:** 1 Department of Materials Science and Engineering, University of Michigan, Ann Arbor, Michigan 48109-2136, USA; 2 Department of Chemical Engineering and Materials Science, University of California—Irvine, Irvine, California 92697, USA; 3 Department of Chemical and Biomolecular Engineering, University of Pennsylvania, Philadelphia, Pennsylvania 19104, USA; 4 Department of Chemistry, University of Pennsylvania, Philadelphia, Pennsylvania 19104, USA; 5 Department of Chemical and Pharmaceutical Sciences, ICCOM-CNR Trieste Research Unit and INSTM, University of Trieste, 34127 Trieste, Italy; 6 Department of Physics and Astronomy, University of California—Irvine, Irvine, California 92697, USA

## Abstract

The exceptional activity for methane combustion of modular palladium–ceria core–shell subunits on silicon-functionalized alumina that was recently reported has created renewed interest in the potential of core–shell structures as catalysts. Here we report on our use of advanced *ex situ* and *in situ* electron microscopy with atomic resolution to show that the modular palladium–ceria core–shell subunits undergo structural evolution over a wide temperature range. *In situ* observations performed in an atmospheric gas cell within this temperature range provide real-time evidence that the palladium and ceria nanoparticle constituents of the palladium–ceria core–shell participate in a dynamical process that leads to the formation of an unanticipated structure comprised of an intimate mixture of palladium, cerium, silicon and oxygen, with very high dispersion. This finding may open new perspectives about the origin of the activity of this catalyst.

## Abstract (web summary)

There is currently renewed interest in the use of core–shell catalysts for methane combustion. Here, the authors perform an *ex situ* and *in situ* electron microscopy study to probe the structural evolution of palladium–cerium dioxide catalytic core–shell subunits over a wide temperature range.

## Graphical abstract

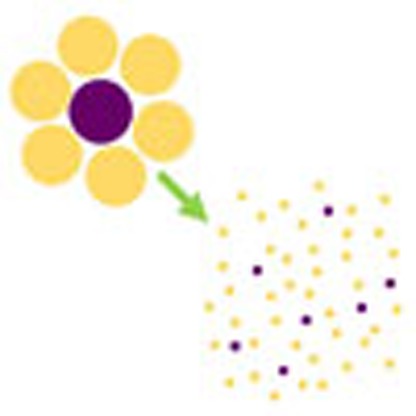

Over the past several years, attention has increasingly been focused on efforts to engineer catalysts at the nanometre scale. These efforts have succeeded in generating a number of interesting and significant results, both in terms of the ability to synthesize new materials and to realize novel functionality. A recent example is the solution-based assembly of units composed of a palladium core and ceria shell (Pd@CeO_2_)^1^, which when homogeneously deposited onto a functionalized alumina support, yield a unique methane combustion catalyst^2^.

Core–shell structures, such as noble metal (Ag, Au, Pd and Pt) particles in a carbon sphere^3^, Pt@CoO yolk/shell nanoparticles^4^, Pt@mSiO_2_ (ref. 5), and Au nanoparticles in hollow ZrO_2_ (ref. 6) and hollow SiO_2_ (ref. 7) spheres, comprise a broad class of nanoengineered catalysts. In cases where a solid metal core is encapsulated in an oxide shell, the goal has usually been to isolate the metal particles from each other, in order to avoid sintering, while keeping active sites accessible. In the case of the Pd@CeO_2_ catalyst^1^,^2^,^8^, another goal was to ensure intimate contact between a central Pd nanoparticle and a surrounding shell, made of comparably sized CeO_2_ nanocrystals, in order to promote more efficient total oxidation of methane. Electron microscopy characterization of the Pd@CeO_2_ catalyst confirmed that a core–shell-like structure had initially been realized, thus apparently accounting for the unique catalytic performance observed.

In an attempt to better understand the connection between structure and activity, we perform a detailed electron microscopy study of supported Pd@CeO_2_, including planar forms of the catalyst, using state-of-the-art *ex situ* and *in situ* transmission electron microscopy (TEM) with sub-angstrom resolution. Our results reveal an unexpected structural transformation that occurs upon air calcination at temperatures between 500 and 800 °C, in which a phase of very highly dispersed palladium and ceria in intimate contact is formed. On the basis of the structure, we propose an alternative explanation for the exceptional catalytic properties.

## Results

### High-surface-area supported Pd@CeO_2_ samples

**Figure 1:**
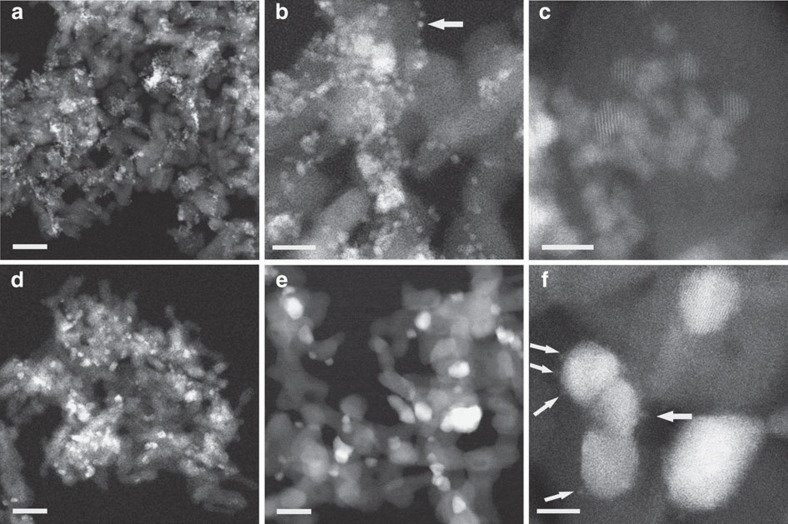
HAADF images of the high-surface-area alumina-supported Pd@CeO_2_ sample. (**a**–**c**) Calcined at 500 °C and (**d**–**f**) calcined at 800 °C, both for 5 h. The arrow in (**b**) indicates an individual 2–3 nm particle. The arrows in (**f**) indicate very small, sub-nm features. Scale bars, 40 nm (**a**,**d**), 15 nm (**b**,**e**), 5 nm (**c**,**f**).

High-angle annular dark-field (HAADF) images, obtained by scanning TEM of a sample of Pd@CeO_2_ supported on high-surface-area Si-functionalized Al_2_O_3_, following air calcination at 500 °C for 5 h, are shown in Fig. 1a–c. Bright features of order 10 nm across, seen in Fig. 1a, are more clearly resolved in images taken at progressively higher magnification (Fig. 1b,c), which show that they consist of clusters of particles, each ∼2–3 nm in diameter. Although the particles usually appear in clusters, of varying size and shape, some are also individually dispersed, such as those indicated by the arrow in Fig. 1b. X-ray energy-dispersive spectroscopy (EDS; Supplementary Fig. 1) showed that clusters typically contain O, Al, Si, Pd and Ce, but the Pd/Ce ratio was found to vary from cluster to cluster. Lattice fringes, when evident, can usually be related to CeO_2_.

For comparison, images obtained from a sample calcined in air at 800 °C for 5 h are shown in Fig. 1d–f. Although the low-magnification images (Fig. 1a,d) look similar, images taken at progressively higher magnification (Fig. 1e,f) show that the bright features are each comprised of only a few (1–3) larger (5–20 nm across) particles. Very small (<1 nm across) features, indicated by arrows in Fig. 1f, were also observed. Pd typically did not appear in EDS spectra, taken at random points (Supplementary Fig. 2), though some very large (tens of nanometre across) palladium particles were found.

### Model planar-supported Pd@CeO_2_ samples

**Figure 2:**
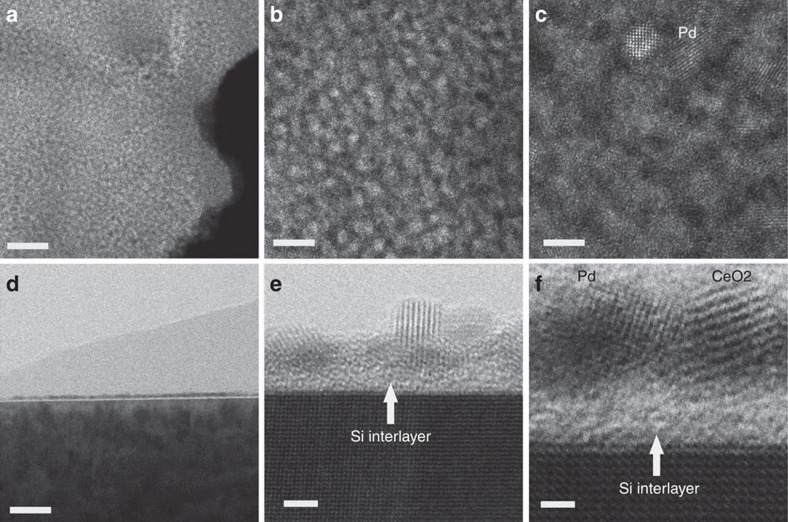
Images of the model planar YSZ-supported Pd@CeO_2_ sample calcined at 500 °C. (**a**–**c**) Plan-view HAADF images and (**d**–**f**) cross-sectional bright-field images. Scale bars, 40 nm (**a**), 8 nm (**b**), 3 nm (**c**), 30 nm (**d**), 2 nm (**e**) and 1 nm (**f**).

In an attempt to simplify the characterization of the system, we examined samples of Pd@CeO_2_ supported on the Si-functionalized surface of single crystals of yttria-stabilized zirconia (YSZ). A low-magnification plan-view image of a sample air calcined at 500 °C for 5 h, shown in Fig. 2a, indicates that the surface is fully coated by a layer of material. Progressively higher magnification images, shown in Fig. 2b,c, reveal a relatively open structure of randomly packed particles, each 2–3 nm across. Lattice spacing, when observed, is usually characteristic of CeO_2_, though spacing corresponding to Pd was occasionally found, as indicated in Fig. 2c. Cross-sectional images, shown in Fig. 2d–f, establish that the layer coating the surface is generally uniform in thickness and consists mainly of single particles of ceria (and some palladium), each ∼2–3 nm across. The 1–2 nm apparent gap between this layer and the YSZ surface is due to the Si interlayer, according to EDS. EDS results, obtained from somewhat thicker regions of the layer, also confirmed the composition of palladium and ceria particles, inferred on the basis of lattice spacing, as shown in Fig. 2f. The valence state of Ce in this sample is 3+, according to electron energy loss spectroscopy (EELS); (Supplementary Fig. 3a,b).

**Figure 3:**
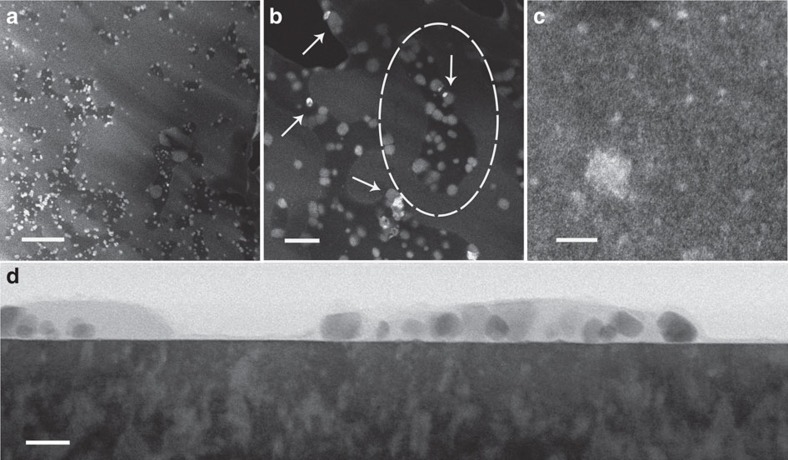
Images of the model planar YSZ-supported Pd@CeO_2_ sample calcined at 800 °C. (**a**–**c**) Plan-view HAADF images and (**d**) cross-sectional bright-field image. The arrows in (**b**) indicate brighter parts of the 10–20 nm features. The region encircled by the oval was used to calculate the number of Ce atoms, as detailed in the Discussion section. Scale bars, 200 nm (**a**), 50 nm (**b**), 8 nm (**c**) and 20 nm (**d**).

Plan-view images of a sample air calcined at 800 °C for 5 h, shown in Fig. 3a–c, provide evidence of two distinct types of features, one with relatively large dimensions, and the other with very small dimensions. The large features are typically 10–20 nm across. EDS confirmed that the brighter parts of these features (some of which are indicated by arrows in Fig. 3b), typically 10 nm across, are comprised of palladium, with ceria comprising the remainder (Supplementary Fig. 4). The number of Pd-containing features is relatively small, as shown in Fig. 3b. In addition to these features, the lower magnification images (Fig. 3a,b) reveal that the surface has one of two distinct appearances, regions of either uniform low or high brightness (in HAADF). The large features are typically found at either the boundaries or entirely within the regions of low brightness, which are rich in Si (Supplementary Fig. 5). The very small features, <1 nm across, are usually uniformly dispersed in areas of high brightness, as shown in Fig. 3c. Cross-sectional bright-field images of this sample, such as that in Fig. 3d, show that most of the 10–20 nm ceria and palladium particles are covered by a thick layer rich in Si, according to EDS. The valence state of Ce in this sample is 4+ (Supplementary Fig. 3c,d).

### Extended calcination and *in situ* observations

**Figure 4:**
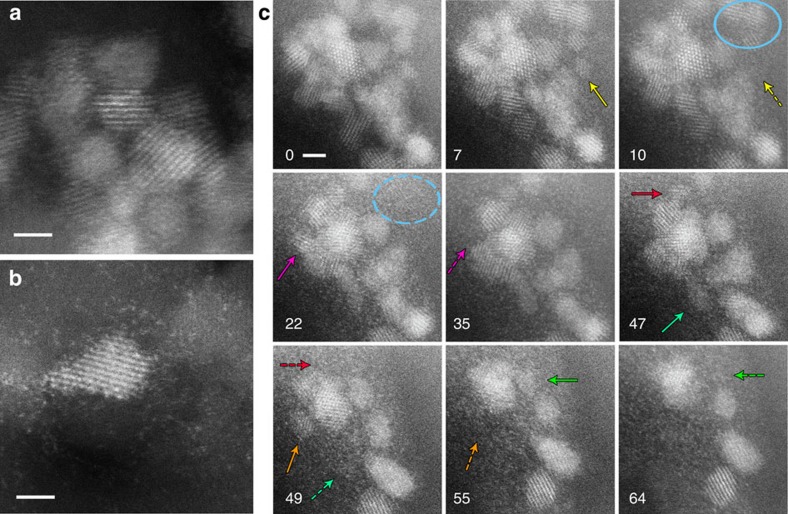
Formation of atomic species during extended calcination. (**a**) Sample calcined at 500 °C (**b**) Sample calcined at 500 °C after 30 min extended *ex situ* calcination at 800 °C, showing that a large number of atomic-scale species were generated. (**c**) Sequential images showing the dissociation of 2–3 nm particles and the formation of atom ‘clouds' during *in situ* calcination at 500 °C in 150-torr O_2_. Elapsed time, in minutes, is indicated on the lower left corner of each image. Arrows and ovals of the same colour are used to indicate the gradual disappearance of the small crystallites. Scale bars, 2 nm.

The presence of very small (1 nm across) features, found in both samples calcined at 800 °C but in neither of the samples calcined at 500 °C, prompted an examination of the effect of a short (30 min) 800 °C *ex situ* air calcination treatment on the high-surface-area supported Pd@CeO_2_ sample that had been calcined at 500 °C. Surprisingly, this treatment caused the entire sample to become covered by a very high population of atomic-scale species that were not initially present. A comparison of the sample before and after the extended calcination is shown in Fig. 4a,b.

In order to better understand this process and the other transformations that occur in samples calcined at elevated temperatures, we conducted an *in situ* calcination treatment with 150 torr of pure oxygen (comparable to the 20% concentration of oxygen in air at atmospheric pressure) in the TEM, using a novel sealed gas cell sample holder. For temperatures below 500 °C, typical clusters of 2–3 nm particles (mostly ceria nanocrystals, as confirmed by the fast Fourier transform pattern shown in Supplementary Fig. 6) remained totally stable, but when the temperature reached 500 °C, a drastic structural transformation, shown in the sequential set of images in Fig. 4c commenced. The atoms on the corners of the smaller and more isolated particles first became mobile and started to leave the cluster. As indicated by the solid and dashed yellow arrows on the images at 7 and 10 min, respectively, a relatively small particle then began to dissociate into a ‘cloud' of atomic-scale species, followed by the dissociation of three more non-overlapping particles, indicated by the circles on images at 10 and 22 min. With increasing time, more crystallites shrank and dissociated, as indicated by other pairs of arrows of the same colour on the sequential images in Fig. 4c, until the majority of particles in this cluster had transformed into atom ‘clouds' after about an hour. Since the alumina surface was Si functionalized, it seems likely that a silica layer is involved in the stabilization of these atomic-scale ‘clouds'. In fact, atom ‘clouds' were not observed in analogous experiments performed on samples that did not contain Si (Supplementary Fig. 7). Although the zones around the particles shown in Fig. 4c gradually become blurred due to formation of the atom ‘clouds', the shrinking particles are undoubtedly ceria, as shown by direct measurement of their lattice symmetry and spacing (Supplementary Fig. 6).

**Figure 5:**
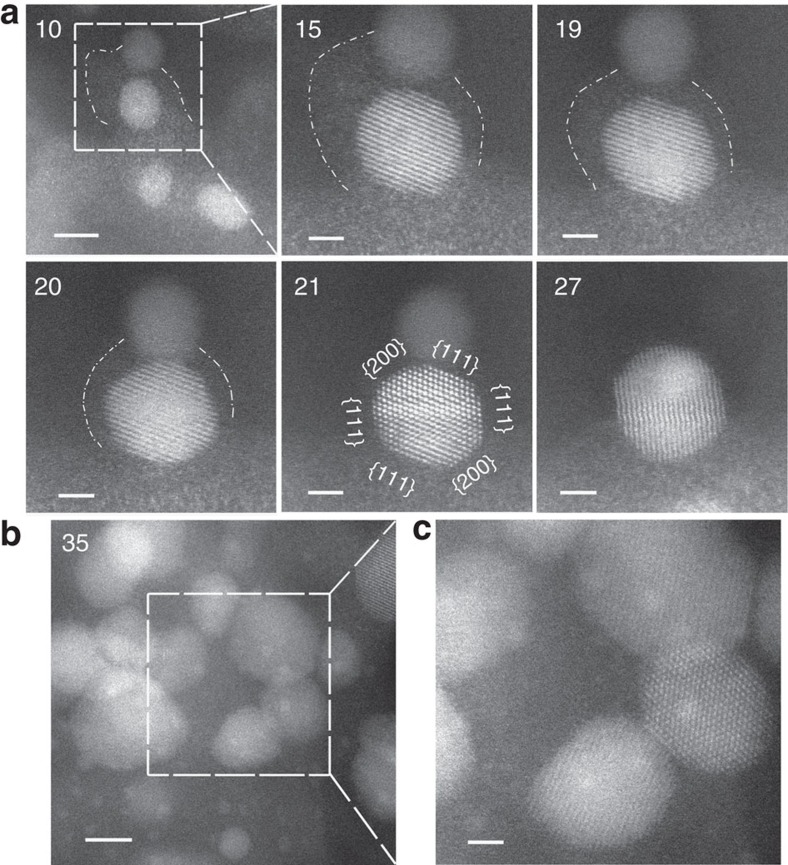
Structural evolution following atom cloud formation. (**a**) Sequential images taken at 650 °C in 150 torr O_2_, showing the gradual disappearance of an atom ‘cloud' accompanied by the growth of a particle in its close vicinity, followed by particle coalescence. The dashed line is used to delineate the periphery of the atom ‘cloud'. The spacing between {111} lattice planes in the larger particle-labelled ‘21', 3.1 Å, confirms it is CeO_2_. (**b**) Appearance of subnanometre features in an image taken at a different area. Elapsed time, in minutes, is indicated on the upper left corner of each image. Scale bars, 5 nm (10), 2 nm (15–27), 5 and 2 nm (**b**).

As the temperature of the sample in the gas cell was increased toward 650 °C, other dynamical processes were also observed, as shown in Fig. 5a. In this example, the two particles in the dashed square are initially of equal size, with the lower one surrounded by a ‘cloud'. The contraction of the ‘cloud', accompanied by the growth of the lower particle (again identified as ceria by its lattice symmetry and spacing) with time can be clearly seen in the sequential images between 15 and 21 min. Concurrently, the particle appears to rotate and change shape in an attempt to minimize its surface energy, until it finally becomes a truncated octahedron bound by eight {111} planes (the lowest surface energy plane) and six {100} planes. This state is well accepted as the most stable shape for CeO_2_ nanoparticles of this size^9^,^10^,^11^. Eventually, the two crystallites coalesce, driven by the tendency to lower overall surface energy. Many such examples of particle coalescence were observed, resulting in CeO_2_ particles of 5–10 nm in diameter. However, very small (1–2 nm) features, similar to those observed in samples after *ex situ* calcination at 800 °C for 5 h, remained throughout the sample, as shown in Fig. 5b. The total heating time of the *in situ* experiment was ∼250 min (Supplementary Fig. 8).

## Discussion

In our attempt to better characterize Pd@CeO_2_ supported on Si-functionalized alumina, the use of a novel *in situ* electron microscopy technique has allowed us to observe the dynamics of structural transformations that occur under an oxidizing atmosphere. *Ex situ* examination of samples calcined for 5 h at 500 °C show that the Pd@CeO_2_ structures are initially formed of a mixture of 2–3 nm palladium and ceria crystallites, generally in close proximity, where Pd is hard to unequivocally discern by lattice spacing or contrast difference. Samples calcined for 5 h at 800 °C contain both large palladium and ceria particles, typically 5–20 nm across, as well as very small entities, <1 nm across, most likely derived from ceria, but possibly also including palladium. A short *ex situ* calcination of the 500 °C sample at 800 °C generates many atomic species, well dispersed on the support.

Observations made on the model planar sample calcined at 800 °C show that large numbers of the very small entities may coexist with the large ceria and palladium particles. These observations also suggest that a highly dispersed form of atomic-scale species may have appeared first, and that the larger particles subsequently grew by consumption of this material in their immediate vicinities. This suggestion is supported by comparison of the number of Ce atoms that would have occupied regions of low brightness, assuming these regions originated from a monolayer of ceria particles, 2–3 nm in diameter, and the number of Ce atoms contained in the large ceria particles within their perimeters. As an example, these numbers agree to within a factor of 2 for the region indicated by the oval in Fig. 3b: 3 × 10^5^ Ce corresponding to a monolayer of ceria particles covering the low brightness region versus 5 × 10^5^ Ce within the large ceria particles.

Such a scenario is actually demonstrated by the *in situ* observations, where dynamic processes were followed in real time: single atoms are released by smaller crystallites at 500 °C in 150 torr oxygen and then stabilize in the form of atom ‘clouds' with low mobility, where silica likely plays some role, either structurally or chemically. However, the ‘clouds' become energetically unfavourable at higher temperature. Atomic species or clusters either add onto some relatively larger particles in close proximity, or nucleate into the very small entities if a large particle is not nearby. The above *in situ* results thus provide insight into the formation of the atomic-scale species and the very small entities observed in samples after *ex situ* calcination. Although the origin of this phenomenon is not yet fully understood, we propose that it essentially reflects the thermodynamic favourability of compound formation between the ceria nanocrystals, which exhibit significant Ce^3+^ character, and the silica in close proximity. The stability of Ce^3+^ in these particles under oxidizing conditions may be related to excess oxygen vacancies in nanoscale ceria, which has been reported by several groups^8^,^12^,^13^,^14^. Similar arguments have been made concerning the surface-to-bulk ratio and valence state change from 4+ to 3+ when the thickness of the wall of ceria nanotubes change from 10 to 5 nm (ref. 14). In any case, initiation of such a reaction between ceria and silica could be facilitated by the lower cohesive energy of the ceria particles, arising both from their high surface area and excess surface oxygen vacancies. It has been shown in other studies that one such compound, Ce_9.33_(SiO_4_)_6_O_2_, tends to gradually decompose back into a mixture of nanoscale ceria and silica as temperature is increased to ∼800 °C in air^15^,^16^. Such decomposition and phase separation is consistent with our observation of the eventual growth of large particles and the appearance of Si-rich deposits around them in the model planar sample calcined at 800 °C.

**Figure 6:**
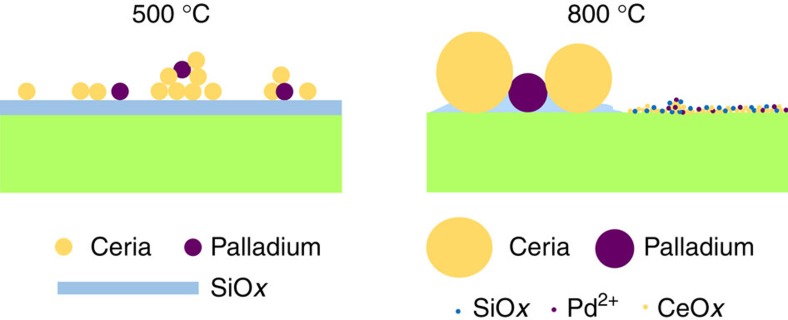
Overall structural transformation. Schematic showing that the original clusters of 2–3 nm particles evolve into a mixture of 5–20 nm particles and a new structure containing Ce, Pd, Si and O in a very highly dispersed form.

While it is clear that the very small entities exist in both powder and model planar catalyst samples, calcined at 800 °C, we propose that a high concentration of the atom-scale species, seen most clearly in the model planar sample, is also present in the powder sample. In contrast, the 2–3 nm ceria and palladium particles do not survive the 800 °C calcination treatment. The structure of supported Pd@CeO_2_ thus evolves as shown in Fig. 6, according to all of our new observations. At the low-temperature extreme, this structure bears some resemblance to the core–shell structure, since 2–3 nm particles of ceria and palladium may be intimately mixed together, but at the high-temperature extreme, which corresponds closely to the calcination treatment used for the previously reported methane combustion experiments (850 °C)^2^, the structure consists of essentially two distinct forms, a mixture of coarse particles of ceria and palladium, that should be characterized by conventional catalytic behaviour, and a new structure in which ceria, palladium and silica are all present in a very highly dispersed form. It would thus appear that the unique catalytic properties arise from the new structure. A question that remains unanswered is the precise composition and structure of the material comprised of the atomic-scale species. It has been reported that Pd-doped ceria–zirconia, or ionic Pd incorporated into the surface of ceria, can inhibit the PdO–Pd transformation^17^,^18^,^19^, which plagues the conventional methane combustion catalyst^2^. It is possible that the material in our newly found structure contains ionic Pd bonded to ceria in such a fashion. Post-reaction TEM examination, or *in situ* TEM studies performed under methane combustion conditions, could provide useful tests of this hypothesis.

## Methods

### Sample preparation

The preparation of Pd@CeO_2_ and its deposition onto Si-functionalized surfaces of Sasol TH100/150 alumina and yttria-stabilized zirconia single crystals was performed as described previously^2^,^8^. All samples were initially calcined in laboratory air for 5 h at either 500 or 800 °C. The short (30 min) 800 °C *ex situ* air calcination treatment, subsequently performed on the high-surface-area sample that had initially been calcined at 500 °C, was done using compressed air.

### TEM specimen preparation

High-surface-area samples were suspended in methanol with sonication and drop cast onto lacy carbon-on-copper grids (Pella). Cross-sectional and plan-view specimens of model planar samples were prepared by standard sample preparation methods. The model planar sample was first glued to a silicon sacrificial layer to protect the surface before diamond saw sectioning into small pieces, followed by mechanical polishing with diamond lapping film and low-angle (3°) Ar ion milling at 3 kV to electron transparency. Plan-view specimens were mechanically polished and ion milled from the substrate side only, thus no surface damage can be created.

### Electron microscopy

Specimens were examined in a spherical aberration (*C*_S_)-corrected JEOL JEM-2100F operated at 200 keV, equipped with EDS detectors and Gatan image filters for EELS acquisition. *In situ* observation was carried out on the same microscope using the Protochips Atmosphere system, which consists of a micro-electrical-mechanical systems (MEMS)-based closed cell, a heating holder and a gas delivery manifold. The sample was situated between two SiN windows, each of 30–50 nm in thickness, with a 5 μm gap in between. The purity of oxygen used in the *in situ* experiment was 99.9995%. All reported temperatures are based on the Protochips calibration.

## Additional information

**How to cite this article:** Zhang, S. *et al*. Dynamic structural evolution of supported palladium–ceria core–shell catalysts revealed by *in situ* electron microscopy. *Nat. Commun.* 6:7778 doi: 10.1038/ncomms8778 (2015).

## Supplementary Material

Supplementary Information Supplementary Figures 1-8.

## References

[b1] Cargnello M., Wieder N. L., Montini T., Gorte R. J. & Fornasiero P. Synthesis of dispersible Pd@ CeO_2_ core−shell nanostructures by self-assembly. J. Am. Chem. Soc. 132, 1402–1409 (2009). 20043676 10.1021/ja909131k

[b2] Cargnello M. . Exceptional activity for methane combustion over modular Pd@ CeO_2_ subunits on functionalized Al_2_O_3_. Science 337, 713–717 (2012). 22879514 10.1126/science.1222887

[b3] Sun X. & Li Y. Colloidal Carbon spheres and their core/shell structures with noble-metal nanoparticles. Angew. Chem. Int. Ed. Engl. 43, 597–601 (2004). 14743414 10.1002/anie.200352386

[b4] Yin Y. . Formation of hollow nanocrystals through the nanoscale Kirkendall effect. Science 304, 711–714 (2004). 15118156 10.1126/science.1096566

[b5] Joo S. H. . Thermally stable Pt/mesoporous silica core-shell nanocatalysts for high-temperature reactions. Nat. Mater. 8, 126–131 (2009). 19029893 10.1038/nmat2329

[b6] Arnal P. M., Comotti M. & Schüth F. High-temperature-stable catalysts by hollow sphere encapsulation. Angew. Chem. Int. Ed. Engl. 118, 8404–8407 (2006). 10.1002/anie.200603507 17109458

[b7] Lee J., Park J. C. & Song H. A nanoreactor framework of a Au@ SiO_2_ yolk/shell structure for catalytic reduction of p-nitrophenol. Adv. Mater. 20, 1523–1528 (2008).

[b8] Adijanto L. . Exceptional thermal stability of Pd@CeO_2_ core-shell catalyst nanostructures grafted onto an oxide surface. Nano Lett. 13, 2252–2257 (2013). 23557343 10.1021/nl4008216

[b9] Zhang F., Jin Q. & Chan S. W. Ceria nanoparticles: size, size distribution, and shape. J. Appl. Phys. 95, 4319 (2004).

[b10] Wang Z. L. & Feng X. Polyhedral shapes of CeO_2_ nanoparticles. J. Phys. Chem. B 107, 13563–13566 (2003).

[b11] Zhang F., Jin Q. & Chan S.-W. Ceria nanoparticles: size, size distribution, and shape. J. Appl. Phys. 95, 4319–4326 (2004).

[b12] Reddy B. M. . Structural characterization of nanosized CeO_2_-SiO_2_, CeO2-TiO_2_, and CeO_2_-ZrO_2_ catalysts by XRD, Raman, and HREM techniques. J. Phys. Chem. B 109, 3355–3363 (2005). 16851365 10.1021/jp045193h

[b13] Tschöpe A. Interface defect chemistry and effective conductivity in polycrystalline cerium oxide. J. Electroceram. 14, 5–23 (2005).

[b14] Han W.-Q., Wu L. & Zhu Y. Formation and oxidation state of CeO_2-*x*_ nanotubes. J. Am. Chem. Soc. 127, 12814–12815 (2005). 16159271 10.1021/ja054533p

[b15] Kępiński L., Wołcyrz M. & Marchewka M. Structure evolution of nanocrystalline CeO_2_ supported on silica: effect of temperature and atmosphere. J. Solid State Chem. 168, 110–118 (2002).

[b16] Rocchini E. . Relationships between structural/morphological modifications and oxygen storage–redox behavior of silica-doped ceria. J. Catal. 194, 461–478 (2000).

[b17] Primavera A., Trovarelli A., de Leitenburg C., Dolcetti G. & Llorca J. Reactivity and characterization of Pd-containing ceria-zirconia catalysts for methane combustion. Stud. Surf. Sci. Catal. 119, 87–92 (1998).

[b18] Priolkar K. . Formation of Ce_1-*x*_ Pd_*x*_O_2-*δ*_ Solid solution in combustion-synthesized Pd/CeO_2_ Catalyst: XRD, XPS, and EXAFS Investigation. Chem. Mater. 14, 2120–2128 (2002).

[b19] Colussi S. . Nanofaceted Pd-O sites in Pd-Ce surface superstructures: enhanced activity in catalytic combustion of methane. Angew. Chem. Int. Ed. Engl. 48, 8481–8484 (2009). 19802862 10.1002/anie.200903581

