# Supplementary material for: Dynamic structural evolution of supported palladium–ceria core–shell catalysts revealed by in situ electron microscopy
Source: Nat Commun. 2015 Jul 10;6:7778. doi: 10.1038/ncomms8778 (PMC4510970; doi:10.1038/ncomms8778)
Supplement: Supplementary Information — Supplementary Figures 1-8. [file ncomms8778-s1.pdf]

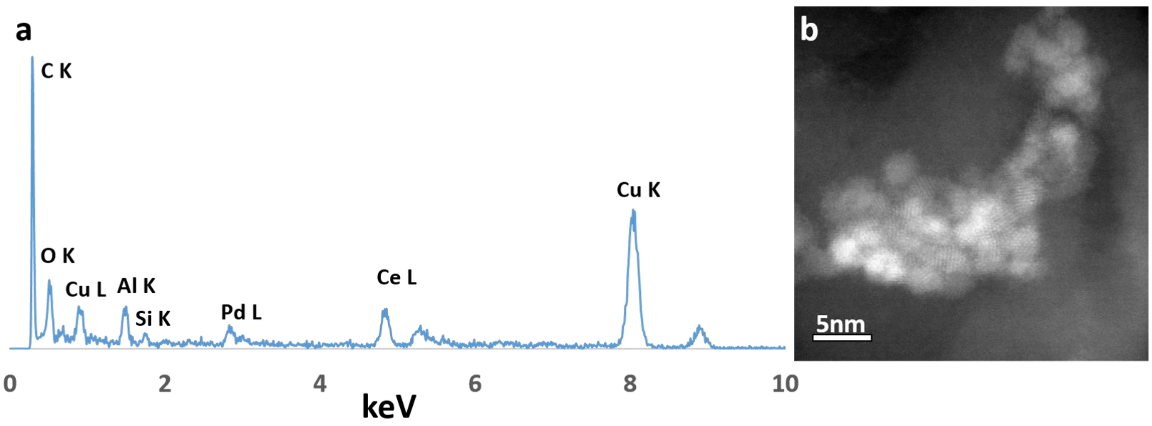


**Supplementary Figure 1| A typical EDS spectrum showing the composition of the high-surface-area Si-functionalized $Al_2O_3$-supported Pd@$CeO_2$ sample calcined at 500 °C.** The spectrum in (**a**) was acquired from the region shown in the HAADF image in (**b**). (The Cu signal arises from the TEM grid, which may also be the source of C.)

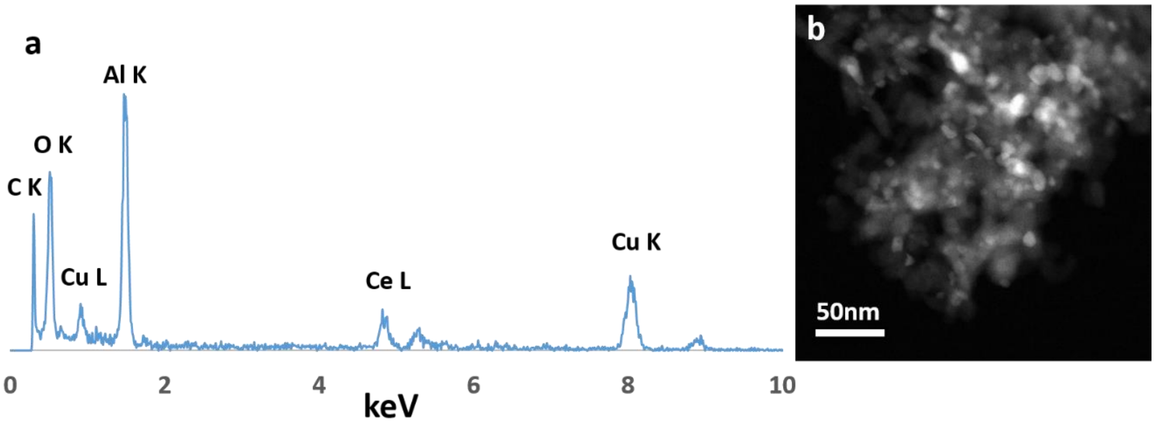


**Supplementary Figure 2| A typical EDS spectrum showing the composition of the high-surface-area Si-functionalized $Al_2O_3$-supported Pd@$CeO_2$ sample calcined at 800 °C.**

The spectrum in (**a**) was acquired from the region shown in the HAADF image in (**b**).

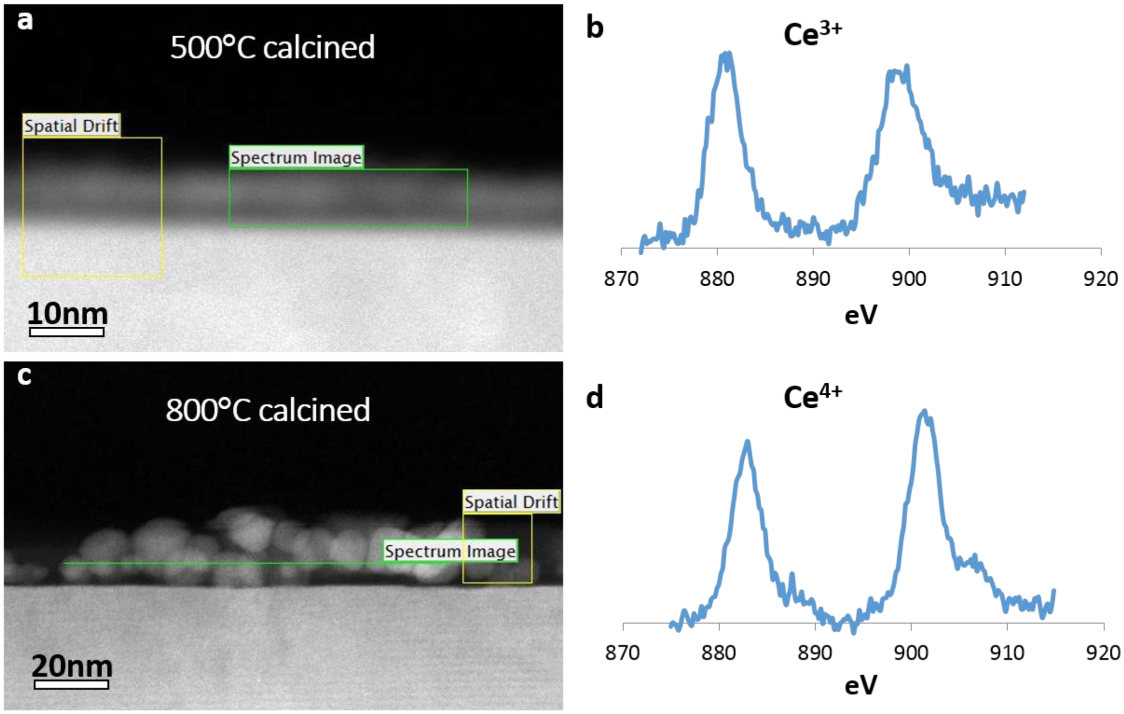


**Supplementary Figure 3| EELS spectra from cross-sectional specimens of model planar Si-functionalized YSZ-supported Pd@$CeO_2$ samples calcined at 500 °C and 800 °C, showing the typical 3+ and 4+ spectral signatures.** The spectrum in (**b**) and (**d**) is extracted from the square or line labeled as "spectrum image" in (**a**) and (**c**), respectively.

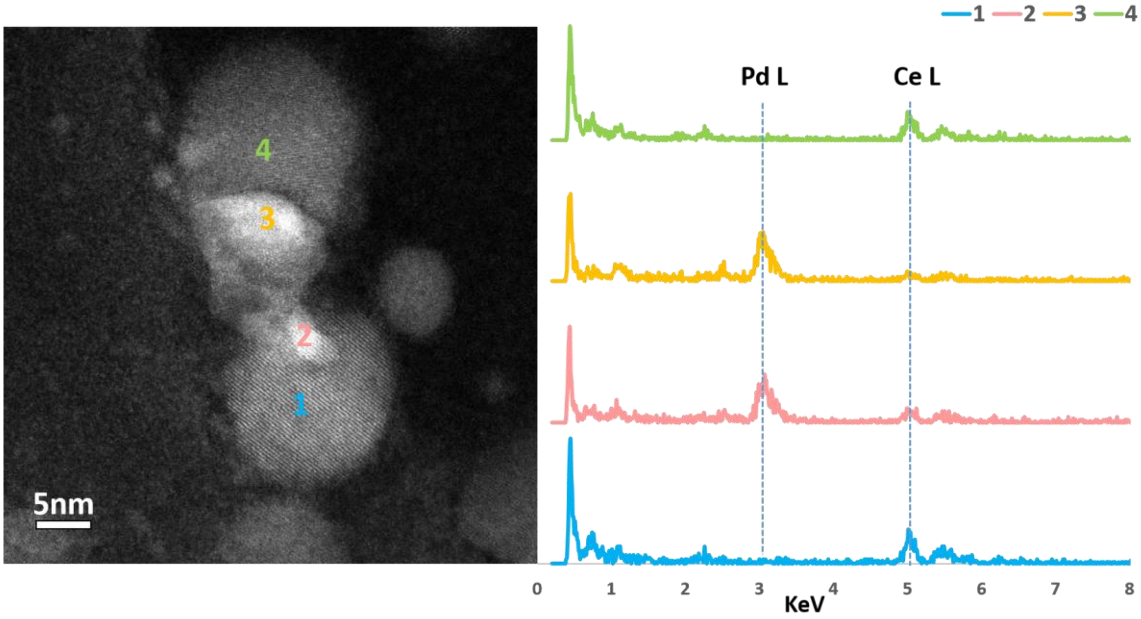


**Supplementary Figure 4 | Typical EDS spectra showing the chemical composition of large particles in a plan-view specimen of the model planar Si-functionalized YSZ-supported Pd@$CeO_2$ sample calcined at 800 °C.** The corresponding spots where the spectra were taken are marked in the HAADF image on the left. Generally, the brighter parts are rich in Pd, while the darker parts are rich in ceria.

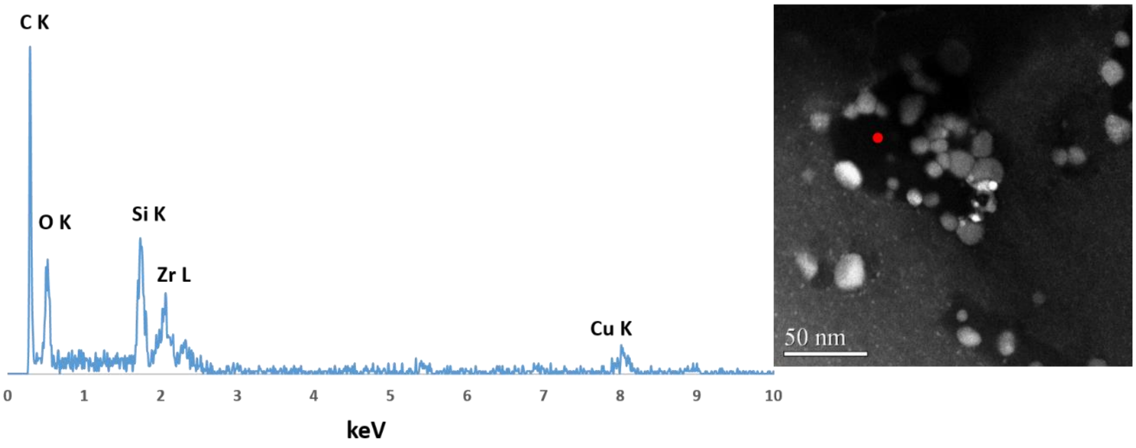


**Supplementary Figure 5|Typical EDS spectrum showing that the low brightness region of the plan-view specimen of the model planar Si-functionalized YSZ-supported Pd@$CeO_2$ sample calcined at 800 °C is rich in Si.** The region where the spectrum was taken is marked by a red spot on the HAADF image at the right. This result corresponds well with similar results from cross-sectional specimens showing that the large particles are covered by a thick layer, rich in Si (Fig. 3d in the main text).

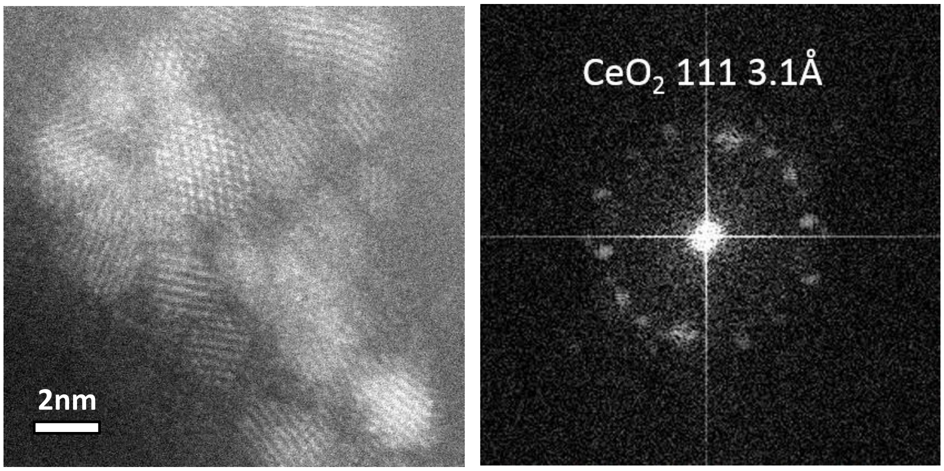


**Supplementary Figure 6| Fast Fourier transform (FFT) of the image labeled as "7" in Fig. 4.** The image labeled as "7" in Fig. 4 is shown on the left, and the FFT, shown on the right, confirms that the lattice planes are (111) from $CeO_2$, with a lattice spacing of 3.1 Å.

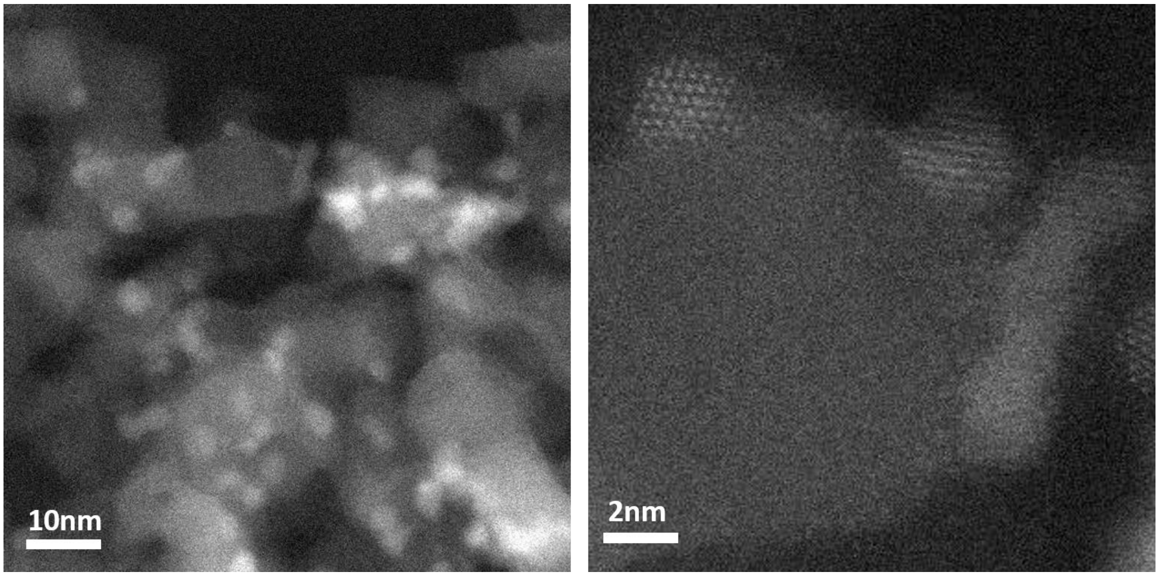


**Supplementary Figure 7| HAADF images acquired from Si-free sample at 500 °C in 150 Torr $O_2$.** Atom "clouds" were not observed around the ceria particles, which are also typically larger than those in the Si-containing sample.

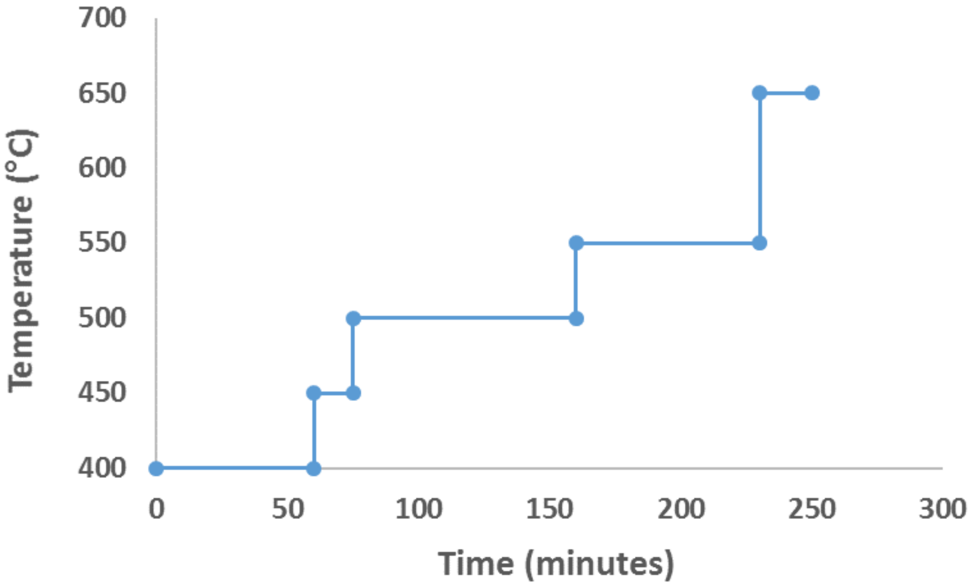


**Supplementary Figure 8| Heating profile during the *in-situ* observations**. The *in-situ* calcination started at a nominal temperature of 400 °C and ended at 650 °C, with total heating time of about 250 min.
